# Modulation of the Anti-Tumor Efficacy of Photodynamic Therapy by Nitric Oxide

**DOI:** 10.3390/cancers8100096

**Published:** 2016-10-20

**Authors:** Albert W. Girotti

**Affiliations:** Department of Biochemistry, Medical College of Wisconsin, Milwaukee, WI 53226, USA; agirotti@mcw.edu; Tel.: +1-414-955-8432; Fax: +1-414-955-6510

**Keywords:** nitric oxide (NO), inducible nitric oxide synthase (iNOS), PDT resistance, post-PDTcell growth/migration/invasion, PDT bystander effects

## Abstract

Nitric oxide (NO) produced by nitric oxide synthase (NOS) enzymes is a free radical molecule involved in a wide variety of normophysiologic and pathophysiologic processes. Included in the latter category are cancer promotion, progression, and resistance to therapeutic intervention. Animal tumor photodynamic therapy (PDT) studies several years ago revealed that endogenous NO can reduce PDT efficacy and that NOS inhibitors can alleviate this. Until relatively recently, little else was known about this anti-PDT effect of NO, including: (a) the underlying mechanisms; (b) type(s) of NOS involved; and (c) whether active NO was generated in vascular cells, tumor cells, or both. In addressing these questions for various cancer cell lines exposed to PDT-like conditions, the author’s group has made several novel findings, including: (i) exogenous NO can scavenge lipid-derived free radicals arising from photostress, thereby protecting cells from membrane-damaging chain peroxidation; (ii) cancer cells can upregulate inducible NOS (iNOS) after a PDT-like challenge and the resulting NO can signal for resistance to photokilling; (iii) photostress-surviving cells with elevated iNOS/NO proliferate and migrate/invade more aggressively; and (iv) NO produced by photostress-targeted cells can induce greater aggressiveness in non-targeted bystander cells. In this article, the author briefly discusses these various means by which NO can interfere with PDT and how this may be mitigated by use of NOS inhibitors as PDT adjuvants.

## 1. Introduction

There is a growing interest in how nitric oxide generated by tumor vascular cells or tumor cells themselves might positively or negatively influence the effectiveness of anti-tumor photodynamic therapy (PDT). There is also an interest in introducing nitric oxide from pharmacologic donors as a possible means of improving PDT outcomes. Each of these aspects with evidence from in vitro (cancer cell) studies and in vivo (animal tumor model) studies will be discussed in this review. Recent evidence that PDT-induced nitric oxide can have far-reaching signaling effects on non-targeted bystander cells will also be discussed. A final section will deal with how the negative effects of nitric oxide on PDT (e.g., resistance to apoptosis and increased growth and migration aggressiveness) can be counteracted with pharmacologic inhibitors of nitric oxide’s enzymatic generation.

## 2. Nitric Oxide and Nitric Oxide Synthases

**Figure 1:**
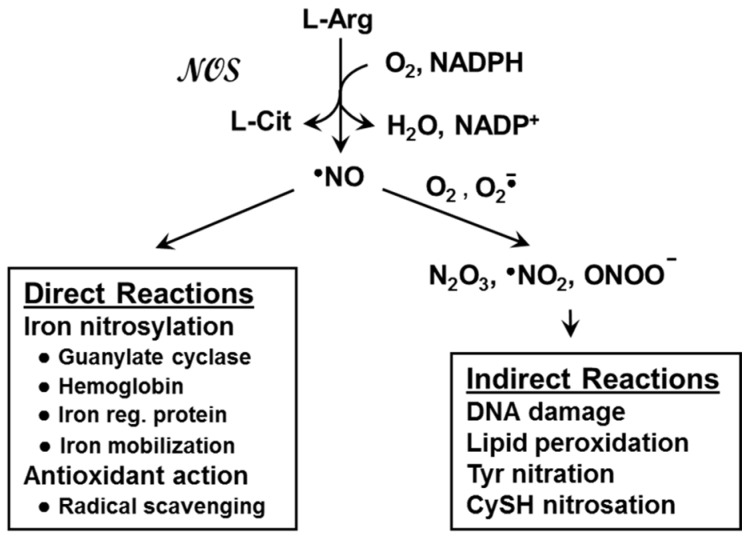
Nitric oxide (NO) generation by nitric oxide synthase (NOS) enzymes, and characteristic direct and indirect reactions of NO.

Nitric oxide (**·**NO or NO for simplicity) is a bioactive free radical that is short-lived (1–2 s in H_2_O), diffuses freely on its own, and tends to partition into hydrophobic milieu such as cell membranes [1,2,3,4]. Naturally occurring NO in mammals is generated by nitric oxide synthase (NOS) enzymes, of which there are three isoforms: neuronal type (nNOS or NOS1), inducible/immune type (iNOS or NOS2), and endothelial type (eNOS or NOS3), based on tissues in which they are most prominent [5,6]. In their active forms, these isoforms exist as 260–320 kDa homodimers, each monomer consisting of a FAD-, FMN-, and calmodulin-containing reductase unit and a tetrahydrobiopterin (BH_4_)- and heme-containing oxygenase unit [6]. nNOS and eNOS require supplemental Ca(II) for optimal activity and operate at low constitutive levels, whereas iNOS does not require additional Ca(II) and can be upregulated to relatively high levels by inflammatory stimuli [6]. NOS enzymes employ NADPH and O_2_ to catalyze the 5-electron oxidation of l-arginine, giving l-citrulline, NADP^+^, and NO as products (Figure 1). Activated nNOS and eNOS, which are often categorized together as constitutive NOS (cNOS), generate NO at low levels (nanomolar range) for less than a minute, whereas activated iNOS generates NO at much higher levels (micromolar range) for hours or even days [4,6]. NOS-derived NO is highly pleiotropic in terms of biological action, playing a role in many different physiological and pathophysiological processes, ranging from vasodilation (eNOS), neurotransmission (nNOS), and antimicrobial activity (iNOS) on the one hand, to chronic inflammation, tumor initiation, and tumor progression on the other, the last three typically associated with iNOS [4,5,6,7].

## 3. Nitric Oxide: Positive and Negative Biological Effects

The chemical biology of NO can be divided into two major categories: direct and indirect activity [3,4]. Direct reactions, which occur at low steady state levels of NO, tend to be regulatory in nature, but in some circumstances may be cytoprotective or even cytotoxic. Indirect reactions, on the other hand, occur at relatively high NO levels and tend to be mainly cytotoxic (Figure 1). At low (nanomolar) levels, NO can react directly with ligated iron in hemoglobin and other heme proteins to give nitrosylated derivatives [3,4,7]. The most common example involves NO produced by eNOS in vascular endothelial cells. Upon diffusion into the sub-endothelial space, this NO nirosylates and activates the heme enzyme soluble guanylyl cyclase (sGC). Activated sGC generates cyclic GMP (cGMP), which promotes blood vessel relaxation and reduction of blood pressure [3,4]. Low level NO may also act as an antioxidant by intercepting and inactivating damaging free radicals, particularly in low polarity lipid membrane environments where NO tends to partition. Studies have shown that NO from a slow release chemical donor such as spermine-NONOate (SPNO, t_1/2_~40 min at 37 °C) can inhibit chain lipid peroxidation in liposomal membranes and cancer cells, protecting the latter against oxidative cell death [8,9,10]. This effect was attributed to interception of chain-propagating lipid oxyl and peroxyl radicals by NO, an example of bimolecular free radical quenching [11].

As indicated above, positive biological effects of low level NO such as vasorelaxation and radical scavenging are sharply contrasted by negative effects brought about by NO at high levels (micromolar range). These effects, which include oxidative damage and cytotoxicity, typically occur at sites of tissue injury or infection and reflect the infiltration of iNOS-expressing macrophages and other immune cells, leading to inflammation. Toxic damage can occur after conversion of NO to a reactive nitrogen species (RNS) such as nitrogen dioxide radical (NO_2_), dinitrogen trioxide (N_2_O_3_), or peroxynitrite (ONOO^−^) (Figure 1). NO_2_ is generated by autoxidation of NO (reaction of 2 NO with O_2_), N_2_O_3_ by reaction of NO with NO_2_, and ONOO^−^ by rapid reaction of NO with superoxide anion radical (O_2_^−^**·**). Superoxide can arise from partial reduction of O_2_ in mitochondria or from activation of NADPH oxidases. Importantly, O_2_^−^**·** can also be produced by NOS enzymes under conditions in which L-arginine or BH_4_ is limiting [4]. Thus, iNOS, for example, may generate O_2_^−^**·** as well as NO under certain conditions, and the resulting ONOO^−^**·** can induce oxidative damage to DNA, membrane lipids, and proteins; tyrosine residues in the latter also susceptible to nitration by ONOO^−^ [1,2,3,4].

It is important to point out that high level NO can be cytotoxic in two different formats with regard to cancer: (i) it may be involved in tumor initiation at sites of inflammation after tissue injury or infection, e.g., in colon cancer associated with chronic colitis; or (ii) it may promote tumor suppression by immune cells, particularly tumor associated macrophages (TAMs), which respond to tumor-associated antigens [3,7,12]. As will be discussed at length subsequently, low level NO produced by tumor cells themselves is a third way that this signaling molecule can affect tumors, but in this case as a pro-survival and pro-growth/mobility mediator.

## 4. Nitric Oxide Signaling in Cancer

**Figure 2:**
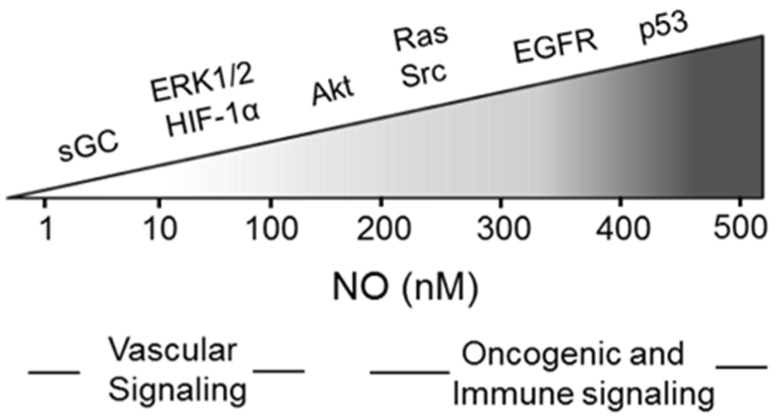
Nitric oxide activation of pro-tumor signaling pathways; NO concentration thresholds for activation of key mediators. (Adapted from Reference [19]).

It is now well established that iNOS-derived NO at low to moderate steady state levels plays a key role in cancer persistence and progression. This may occur via activation of oncogenic signaling pathways or inhibition of tumor-suppressing pathways, and for several different cancers, iNOS has been described as having oncoprotein characteristics [13,14,15,16,17,18]. iNOS/NO expression in cancer cells can activate signaling cascades mediated by proteins such as sGC/cGMP, hypoxia-inducible factor-1α (HIF-1α), epidermal growth factor receptor (EGRF), phosphoinositide-3-kinase/Akt (PI3K/Akt), and extracellular signal-regulated kinases-1/2 (ERK1/2). These proteins are all salutary with respect to cancer cell survival, proliferation, and migration. Based on numerous lines of evidence, it appears that there is a gradation of NO level effectiveness in activating these different pathways (Figure 2). For example, sGC/cGMP, which stimulates pro-growth protein kinase-G (PKG), is activated by NO in the low nM range. However, EGFR, lying upstream of PI3K/Akt and other mediators, requires upwards of 300 nm NO for activation, which reflects phosphorylation of a specific tyrosine residue [3,7]. It is important to add that iNOS/NO and the tumor suppressor p53 have been shown to operate in opposing fashion in many tumors. Thus, wild-type p53 can either block iNOS transcription or bind to iNOS enzyme and reduce its activity [19,20,21]. On the other hand, NO has been shown to modify wild type p53 in breast cancer cells, reducing its pro-apoptotic activity [22]. Moreover, cancer cells with dysfunctional mutant p53 have been shown to express higher levels of iNOS than non-mutant counterparts, and this correlated with an increased proliferation rate and expression of angiogenic factors [23]. It is not surprising, therefore, that the expression level of iNOS in many different tumors is now considered as a reliable prognostic indicator of survival chances, patients with relatively high levels given the poorest chances [2,7].

A question of major interest at this point is: How does NO at relatively low to moderate levels function as a pro-survival or anti-apoptotic signaling molecule in biochemical terms? One distinct possibility relates to modification of cysteine sulfhydryl (–SH) groups of specialized cysteine residues in key effector proteins. These –SH groups typically have lower than normal pK_a_ values (e.g., <6 vs. ~8.5), making them more susceptible to NO-mediated modification, which is defined as S-nitrosation, not “S-nitrosylation”, which is a common error [4]. NO itself cannot add directly to a thiolate (–S^−^) group to produce an S-nitroso (SNO) group. However, it can do so if the thiolate is oxidized to a thiyl radical (–S**∙**) or if NO is oxidized to a nitrosonium ion (NO^+^), neither of which is prominent in most biological systems [4]. However, the oxidized NO intermediate, N_2_O_3_, can readily add to thiolate groups to give SNO derivatives [4,24]. S-nitrosated proteins can initiate signaling cascades which subside when the SNO groups are removed, e.g., by glutathione or thioredoxin [25]. Based on published data for a variety of cell systems, potential effector protein targets for SNO modification include: (i) pro-apoptotic mitogen-activated protein kinases (MAPKs) such as JNK and ASK1, which become inhibited [26,27]; (ii) procaspase-9, whose cleavage to apoptosis-initiating caspase-9 is inhibited [28]; (iii) anti-apoptotic Bcl-2, whose proteosomal degradation is inhibited [29]; (iv) oncogenic EGFR, which is activated due to specific phospho-tyrosine formation [30]; (v) tumor suppressor PTEN, which is inactivated as a negative regulator of PI3K/Akt [31]; and (vi) phosphatase MKP-1, a de-activator of pro-apoptotic phospho-JNK and phospho-p38; its proteasomal degradation is inhibited by SNO-modification [32]. S-nitroso formation is not the only way by which NO or RNS can modify effector proteins. Tyrosine nitration (Tyr-NO_2_), for example, can also occur, but this typically requires much higher NO fluxes than apply for SNO-modification, and highly reactive RNS such as ONOO^−^ are typically involved [3,7].

## 5. Nitric Oxide and PDT: Early Studies

It is now clear that low level endogenous NO not only promotes survival, angiogenesis, and expansion of many tumors, but also resistance to various therapies. For example, Saleem et al. [33] found that apoptosis of non-small cell lung carcinoma cells after exposure to ionizing radiation was stimulated by a non-specific NOS inhibitor. This correlated with down-regulation of hypoxia inducible factor-1α (HIF-1α) and pyruvate dehydrogenase kinase-1 (PDK-1), both of which are associated with tumor survival/progression [33]. Thus, it appeared that NOS/NO, acting through HIF-1α and PDK-1, protected cells against radiation-induced apoptosis. In a more recent study, Matsunaga et al. [34] showed that exposing lung carcinoma cells to cis-platin (CPPD) resulted in an upregulation of iNOS mRNA and protein. iNOS-derived NO appeared to protect against CPPD cytotoxicity because the latter was exacerbated by a NOS inhibitor, but suppressed by supplemental NO from a chemical donor [34].

Although the possibility of NO-mediated resistance to PDT (or perhaps even involvement in PDT) received some serious attention prior to ca. 2000, it was surprisingly little compared with other anti-tumor modalities. As one example, Gupta et al. [35] reported that epidermoid cancer A431 cells rapidly upregulated cNOS and NO after a Pc4-sensitized photodynamic challenge; apoptotic cell death was observed and was attributed to NO-derived cytotoxic species such as ONOO^−^. However, the more plausible possibility that NO was acting cytoprotectively, but was ultimately overwhelmed by photooxidative pressure was not considered [35]. In a more recent study, Gomes et al. [36] showed that AlPcS_2_-sensitized photokilling of lymphoblastoid cells was suppressed by NO from chemical donors. A sGC inhibitor, ODQ, partially reversed this suppression, suggesting involvement of cGMP-dependent PKG in the NO protective effect [36]. This may have been a unique case for these lymphoblastoic cells because most non-PDT-related studies relating to NO-induced resistance and studies by the author’s group (see below) revealed non-sGC/cGMP-mediated effects [3,7].

The first studies to determine how endogenous NO might affect PDT efficacy at the in vivo level were carried out around the same time by two separate groups using mouse syngeneic tumor models. Henderson et al. [37] found that the outcome of Photofrin^®^-sensitized PDT (QLT Photo-Therapeutics, Vancouver, BC, Canada) for radiation-induced fibrosarcoma (RIF) tumors in mice was much improved when the non-specific NOS inhibitor L-NNA was administered before and after irradiation. Shortly before and after this, Korbelik et al. [38,39] showed that the Photofrin^®^-PDT cure rate for RIF and SCCVII tumors, but not EMT6 or FsaR tumors, was significantly improved when either L-NNA or L-NAME (another NOS inhibitor) was introduced immediately after irradiation. Of special interest was the evidence that RIF and SCCVII tumors produced NO at a much greater constitutive rate than EMT6 or FsaR counterparts, thus accounting for the greater responsiveness of the former two to inhibitors [39]. As anticipated, RIF and SCCVI tumors with relatively high NO output, exhibited lower intrinsic sensitivity to PDT than EMT6 and FsaR tumors, suggesting that output measurements might be used as prognostic indicators of PDT efficacy [39]. More recently, Reeves et al. [40], also studying mouse syngeneic tumors, but in this case sensitized with 5-aminolevulinic acid (ALA)-induced protoporphyrin IX (PpIX), confirmed that tumor-generated NO exerted a negative effect on PDT efficacy. It was posited that knowledge of NO yield could be used to predict PDT efficacy and whether NOS inhibitors might improve this [40]. Each of the cited studies [37,38,39,40] reached essentially the same conclusion relating to the tumor vasculature, viz. that vasodilation due to endothelium-derived NO (presumably eNOS-generated NO) was counteracting PDT’s well known tumor-abating vasoconstrictive effects. Although this early work [37,38,39] was groundbreaking in dealing with the effects of endogenous NO on PDT outcomes in vivo, it left many questions unanswered, including: (i) whether the tumor itself (i.e., in addition to or instead of endothelium) can supply NO that might antagonize PDT; (ii) if so, which NOS isoform plays the major role; (iii) whether a basal level of NOS/NO is sufficient for PDT resistance or whether upregulation due to photodynamic stress plays a more important role; and (iv) the types of NO-mediated resistance signaling pathways that might be activated by PDT stress. The following sections will describe more recent studies that have addressed such questions.

## 6. Nitric Oxide and PDT: Relatively Recent Studies

Since ca. 2002, studies in the author’s laboratory have focused on basic mechanisms by which NO can thwart the cytotoxic effects of photodynamic stress on tumor cells. Most of this work has involved ALA-induced PpIX as the sensitizing agent and conditions were established whereby PpIX was either localized to mitochondria (where it originates via the heme biosynthetic pathway [41]) or allowed to diffuse to the plasma membrane and act mainly from there [42]. These two arrangements allowed us to mimic the effects of many pre-existing sensitizers which, when applied to cancer cells, can localize at distinct intracellular locations, including mitochondria vs. plasma membrane [43,44]. In initial work, we examined the effects of low level NO from exogenous donors on cell resistance to PDT-like photokilling, whereas in more recent work, we focused on endogenous NO from one or more NOS donors. Some results of these studies are discussed in the following sections.

### 6.1. Breast Cancer Cells

In model studies dealing with how NO might modulate PDT efficacy, considerable attention has been given to a human breast cancer subline, COH-BR1, which is characterized by its inability to express glutathione peroxidase type-4 (GPx4). GPx4 is a selenoenzyme that can catalyze the reductive detoxification of lipid hydroperoxides (LOOHs) in biological membranes, including phospholipid- and cholesterol-derived species (PLOOHs and ChOOHs) [42]. During singlet oxygen (^1^O_2_)-mediated PDT, these species will arise from direct addition of ^1^O_2_ to unsaturated membrane lipids. In the absence of reducing agents and/or properly ligated redox metal ions such as Fe(III) or Cu(II), LOOHs will accumulate with continuous ^1^O_2_ generation, resulting in membrane perturbation and loss of function. However, if Fe(II) or Cu(I) is available and can be readily recycled to this form, LOOHs can undergo iron-mediated one-electron reduction to lipid oxyl radical (LO**∙**) intermediates. The latter, or more likely, their epoxy-allylic peroxyl radical (OLOO**∙**) derivatives can initiate damaging chain lipid peroxidation by abstracting hydrogen atoms from proximal unsaturated lipids [11]. In the process, new LOOHs are generated, which, like the primary or “priming” LOOHs, are also susceptible to one-electron reduction. If not curtailed in some way, primary and secondary (propagative) LOOH turnover will expand and exacerbate membrane damage and dysfunction [11]. One prominent means of curtailment is by GPx4-catalyzed two-electron reduction of LOOHs to redox-inert alcohol (LOH) species. It is now well established that by reducing primary PLOOHs and ChOOHs generated by photooxidative stress, GPx4 should contribute significantly to tumor cell resistance to PDT [45]. It is now clear that NO can also contribute to resistance, but by entirely different mechanism(s), as discussed below.

As indicated in a previous section, low flux NO may function as a chain-breaking antioxidant by intercepting and inactivating lipid-derived free radicals [9,10]. Using breast cancer COH-BR1 cells, Niziolek et al. [46,47] were the first to investigate this in the context of ALA-PDT. Cells were sensitized with ALA-induced PpIX, which was allowed to diffuse from mitochondria to plasma membrane. When irradiation was carried out in the presence of a catalytic iron chelate, chain lipid peroxidation induced by one-electron reduction of ^1^O_2_-generated LOOHs occurred and the cells died mainly by plasma membrane-rending necrosis [47]. Chain peroxidation and necrosis were strongly suppressed by SPNO-derived NO acting as a LO∙/LOO**·** quencher. These were described as immediate cytoprotective effects of NO because active SPNO had to be present from the start of irradiation. Subsequent work revealed that NO could also protect cells in a delayed manner analogous to pre-conditioning [48,49]. For example, when COH-BR1 cells were first treated with SPNO at an innocuous concentration (e.g., 0.2 mM) and then, after a 20 h delay, sensitized with disseminated PpIX and irradiated, a marked resistance to necrosis was again observed, compared with a non- or spent-SPNO control [48,49]. These effects could be recapitulated when cells were co-incubated with iNOS/NO-expressing RAW 264.7 macrophages ~12 h after their activation; L-NAME attenuated the observed photoresistance, confirming that NO was also acting cytoprotectively in this system. Heme oxygenase-1 (HO-1) and ferritin (both known to have antioxidant properties) appeared to play a major role in NO’s delayed protective effects. The levels of both proteins were substantially elevated 20 h after cells were exposed to SPNO or pre-activated macrophages [48,49]. A reduced level of redox-active cytosolic free iron was also observed (as expected from ferritin induction) and the observed drop in membrane-damaging lipid peroxidation was attributed to this [49]. It is clear from these findings that NO’s delayed (or “NO-then”) cytoprotective effects were based on signaling activity, whereas NO’s immediate (or “NO-now”) effects required its presence when cells were photochallenged. This is a good example of the mechanistic diversity of NO as a direct and indirect antioxidant.

**Figure 3:**
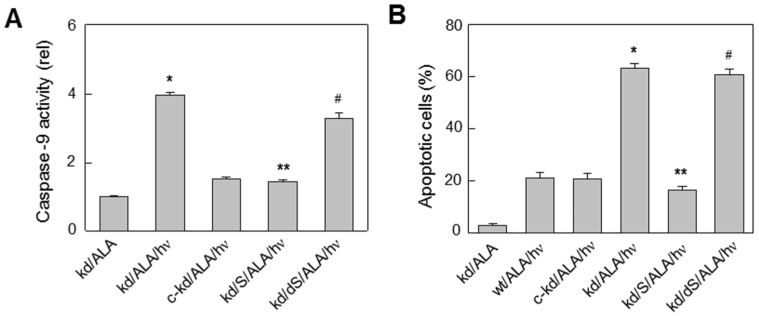
Effect of iNOS knockdown on extent of caspase-9 activation and apoptosis of ALA/light-challenged COH-BR1 cells. Wild-type (wt), shRNA-induced knockdown (kd), and control knockdown (c-kd) cells were pre-incubated with 1 mM ALA (45 min) and irradiated (1 J/cm^2^) in the absence vs. presence of 0.1 mM SPNO (S) or decomposed SPNO (dS), added 10 min before irradiation: (**A**) Caspase-9 activity at 2 h post-hν; and (**B**) Hoechst-assessed apoptosis at 20 h post-hν. Plotted values: means ± SD (n = 3); * *p* < 0.001 compared with c-kd/ALA/hν; ** *p* < 0.001 compared with kd/ALA/hν; ^#^ *p* < 0.01 compared with kd/S/ALA/hν. (Reproduced from Reference [51], with permission).

A major advance was made when the author’s group discovered that breast COH-BR1 cells can provide their own cytoprotective NO in response to photodynamic stress. When cells were sensitized in mitochondria with ALA-induced PpIX and irradiated with broad band visible light, a striking upregulation of immunodetectable iNOS protein was observed during post-irradiation incubation, which reached 2-3-times the dark control level after 2 h and persisted for at least 20 h [50,51]. The other NOS isoforms, nNOS and eNOS, were either undetectable in these cells or did not increase from a low basal level. The photodynamic conditions used were relatively modest (e.g., light fluence ~1 J/cm^2^), resulting in 20%–25% intrinsic apoptotic cell death 20 h after irradiation compared with <5% for light-only or ALA-only controls. Importantly, when an iNOS competitive inhibitor (1400 W) or a NO scavenger (cPTIO) was present during and after irradiation, the extent of caspase-9 activation and apoptosis increased substantially (Figure 3) [50,51]. This also occurred when cells with shRNA-induced iNOS knockdown were photodynamically challenged; in this case, SPNO-donated NO effected a complete rescue (Figure 3) [51]. Taken together, these findings indicate that NO from basal and/or stress-induced iNOS was signaling for increased resistance in COH-BR1 cells.

Progress has been made in defining the signaling events involved in iNOS induction and NO-mediated hyper-resistance in ALA/light-treated cells. For iNOS induction, immunocytological and immunoblot analyses indicated that subunit p65 of transcription factor NF-κB moved from cytosol to nucleus of COH-BR1 cells after a photochallenge [52]. Bay11-7082, which inhibits Iκκ (the kinase that activates NF-κB by phosphorylating its inhibitory subunit IκB) did three things: (i) suppress p65 translocation to nucleus; (ii) suppress iNOS upregulation after an ALA/light chellenge; and (iii) increase the extent of apoptosis after challenge [52]. This evidence established a direct link between photostress-activation of NF-κB, iNOS transcription/translation, NO upregulation, and apoptosis resistance. Other data revealed that the tumor-promoting kinase Akt/PKB was rapidly activated by phosphorylation in ALA/light-challenged cells. Wortmannin, an inhibitor of PI3K, which activates Akt via membrane phosphatidylinositol triphosphate (PI3P) formation, blocked Akt activation and iNOS upregulation in photostressed COH-BR1 cells while simultaneously increasing the extent of apoptosis [52]. These findings implicate activation of upstream Akt in the activation NF-κB (probably by Akt-mediated Iκκ phosphorylation), resulting in greater iNOS transcription. Related work revealed that NO-dependent resistance in COH-BR1 cells was not mediated by sGC/cGMP (e.g., via PKG activation) because the sGC inhibitor ODQ failed to elevate the extent of ALA/light-provoked apoptosis, nor did supplementation with 8-Br-cGMP reduce it [52]. On the other hand, the mitogen-activated protein kinase (MAPK) enzymes JNK and p38 were rapidly, but transiently activated by phosphorylation after a photodynamic challenge; both the extent and duration of these activations were enhanced by 1400 W treatment or iNOS knockdown, suggesting that NO was acting in opposing fashion, i.e., as a promoter of apoptosis resistance. Consistent results were obtained when other effector proteins in COH-BR1 cells were interrogated. For example, pro-apoptotic Bax was upregulated after ALA/light stress, whereas anti-apoptotic Bcl-xL was down-regulated, and each of these responses was significantly enhanced by 1400 W or cPTIO [53]. Moreover (and as anticipated), the indicated post-irradiation changes in Bax and Bcl-xL expression were attenuated by SPNO, which supplied NO exogenously [54]. These findings added further support to the notion that low level NO can signal for PDT resistance.

Recent work carried out in the author’s laboratory provided additional supporting evidence at the in vivo (animal model) level. Immune-deficient female mice bearing human breast carcinoma MDA-MB-231 tumor xenografts were subjected to ALA-PDT using a 633 nm light source. Treated animals exhibited a significant reduction in tumor growth relative to light-only controls over a 12-day post-irradiation period, and 1400 W reduced growth even further [55]. Analysis of post-PDT tumor samples revealed a strong elevation in iNOS and NO-derived nitrite levels. This is the first known evidence for NO resistance to PDT directed against a human tumor in vivo. In all previous animal model PDT studies involving NO, immune-normal mice bearing syngeneic tumors were used [37,38,39,40]. It is important to point out that in immunocompetent animals, an acquired immunity due to photodynamic damage can contribute significantly to tumor eradication by PDT [43,44]. However, NO is known to be immunosuppressive [2,7,12] and this would tend to mitigate the anti-tumor immunologic effects elicited by PDT. Thus, NO can antagonize PDT in different ways and the underlying mechanisms are still not fully understood.

### 6.2. Prostate Cancer Cells

Bhowmick and Girotti [56] and Fahey and Girotti [57] recently demonstrated that two prostate carcinoma cell lines, PC3 and DU145, could also exploit iNOS-generated NO for resistance to PDT cytotoxicity. In these studies, ALA-treated PC3 cells with mitochondrial PpIX responded to a modest light dose with a rapid and prolonged increase in iNOS level, which reached 10–12-times the very low background or light-only control level within 2 h after irradiation. This remarkable and highly reproducible upregulation was much greater than that observed with COH-BR1 cells, which expressed greater basal iNOS [50,51]. DU145 cells also upregulated iNOS under photostress, but less extensively than PC3 counterparts [57]. It is important to note that all experiments described up to this point [50,51,52,53,54,55,56,57] started with cells at confluence no greater than ~60%. At significantly higher confluences, e.g., >90%, the stress from inter-cell contact was sufficient to elevate iNOS [58] and this could have resulted in an under-estimation of the elevation caused by photostress. The very robust induction of iNOS in ALA/light-stressed PC3 cells suggests that greater apoptotic resistance must have been mainly due to induced enzyme, basal level enzyme contributing relatively little. Of added interest was the finding that PC3 remaining alive after a modest ALA/light challenge continued to proliferate, but not at the control (ALA-only or light-only) rate. Instead, these cells grew at a significantly greater rate, which could reach >3-times the background rate from 24 to 48 h after irradiation, DU145 cells showing similar behavior [55,56]. These striking post-irradiation growth spurts were greatly suppressed by 1400 W or cPTIO, indicating that iNOS/NO was playing a dominant driving role [56]. Another notable discovery was that cells surviving photodynamic stress exhibited significantly greater motility in terms of migration and invasion, as determined by gap-closure (wound-healing) and trans-membrane (Boyden chamber) assays, respectively [55]. Once again, 1400 W and cPTIO exerted strong inhibitory effects, implying iNOS/NO involvement. Further supporting evidence was obtained by showing that NO from a slow-releasing donor, DETA/NO, mimicked the effects of photostress by stimulating PC3 proliferation, migration, and invasion, at least at low concentrations. Thus, 10 µM DETA/NO acted as described, but 100 µM inhibited motility and led to growth arrest; see Section 3 [56]. By catalyzing the degradation of extracellular matrix (ECM) proteins, matrix metalloproteinases (MMPs) are known to play a key role in cancer cell migration, invasion and metastasis [59]. MMP-9, for example, is found in found in many different tumors, including prostate tumors, and its elevated expression usually correlates with poor prognosis [60]. Using in-gel zymography to monitor MMP-9 activity in ALA/light-challenged PC3 cells, Fahey and Girotti [55] found that activity increased significantly after photostress, and in light-fluence-dependent as well as 1400 W- or cPTIO-inhibitable fashion. Related work involving immunoblotting showed that tissue inhibitor of metalloproteinase-1 (TIMP-1) was down-regulated after photostress, whereas the α6 and β1 integrins were upregulated, each response being suppressed by 1400 W or cPTIO. As described in this section, increased NO-dependent migration/invasion aggressiveness of surviving cells is a newly discovered negative consequence of photodynamic stress, which, if occurring during clinical PDT could promote tumor metastases to distant sites.

### 6.3. Glioblastoma Cells

**Figure 4:**
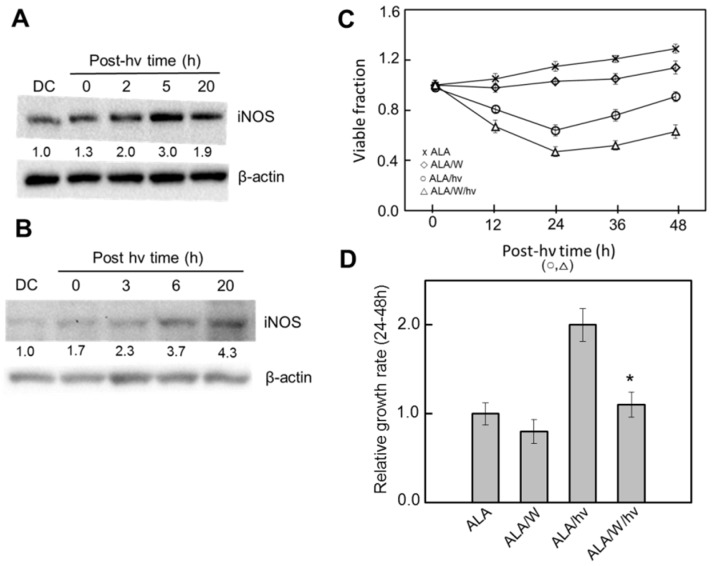
Effects of ALA/light treatment on post-irradiation iNOS expression, viable cell count, and growth rate of glioblastoma cells: (**A**) iNOS immunoblot for U87-MG cells; and (**B**) iNOS immunoblot for U251-MG cells; DC, dark control; numbers below bands are intensities normalized to actin and relative to DC. (**C**) Loss of U87-MG viability after ALA/light in absence vs. presence of 1400 W (W), then subsequent proliferation of surviving cells (24–48 h). (**D**) Quantified growth rate (24–48 h) for each condition in panel C; plotted values are means ± SD (n = 3); * *p* < 0.01 compared with ALA/hν. (Reproduced from Ref. [61], with permission).

Recent studies by Fahey et al. [61] have shown that iNOS/NO also plays a key stimulatory role in resistance of glioblastoma cells to photokilling and in aggressiveness of surviving cells. Glioblastomas are the most difficult to treat and most lethal of all brain malignancies, ultimately resulting in more than ten thousand patient deaths per year in this country [62]. Despite many advances, chemotherapy and radiotherapy have proven to be inadequate for improving longevity in glioblastoma patients and median survival time after diagnosis has been little more than one year [62]. However, PDT, particularly ALA-based PDT, has shown considerable promise in this regard [63,64,65,66], resulting in significantly longer survival compared with other modalities, although there is much room for further improvement. A large part of this improvement could come with the recognition that many human gliomas, including glioblastomas, express significant iNOS and that iNOS-generated NO plays a major role in tumor survival and progression [67,68]. Using human glioblastoma U87-MG and U251-MG cells sensitized with ALA-induced PpIX in mitochondria, Fahey et al. [61] showed that these cells added substantially to their pre-existing iNOS after being irradiated, i.e., protein levels increased up to 4-fold over a 20 h post-irradiation period (Figure 4A,B).

As expected [69], U87-MG cells also expressed significant nNOS, but unlike iNOS, its level did not change after a photodynamic challenge [61], suggesting that only constitutive nNOS/NO could possibly contribute to any cytoprotective or pro-growth effect observed after photostress. Signals from pre-existing NO, as detected with the fluorophore probe DAF-2, increased progressively in U87 cells after ALA/light exposure, and 1400 W markedly reduced their intensity, implying iNOS (but not nNOS) derivation. Another key finding was that the extent of photostress-induced U87 or U251 apoptosis was strongly enhanced by 1400 W or cPTIO, suggesting that NO from stress-induced (and possibly pre-existing) iNOS was signaling for increased resistance [61]. Any possible protective role for nNOS/NO was again ruled out because 1400 W is highly specific for iNOS and, besides, iNOS generates NO at a considerably higher rate than nNOS. Of added importance are the findings that U87 cells which survived ALA/light treatment exhibited a striking boost in the rate of proliferation, (Figure 4C,D), migration, and invasion, each of which was counteracted by 1400 W or cPTIO. This pointed to iNOS/NO as a dominant determinant of increased aggressiveness similar to that observed for photostressed breast and prostate cancer cells [50,51,52,56,57]. Although low flux NO from constitutive iNOS has been widely reported to support survival, expansion, and therapeutic drug resistance of malignant gliomas, the findings reviewed here are entirely novel with regard to anti-glioma PDT.

## 7. Duality of iNOS/NO-Mediated Signaling in PDT

Other investigators have recently addressed similar questions dealing with how NO can influence PDT outcome. For example, Rapozzi et al. [70,71,72] reported that endogenous iNOS/NO can modulate pheophorbide a-sensitized photokilling of melanoma cells in distinct ways, depending on the intensity of photodynamic action. Thus, low level NO induced by modest photooxidative pressure signaled for cytoprotection via upregulation of anti-apoptotic NF-κB and Snail (zinc finger protein SNAI1), but down-regulation of RKIP (Raf kinase inhibitor protein). In contrast, a higher level of NO induced by greater photooxidative pressure signaled for less protection (greater apoptosis) via down-regulation of NF-κB and Snail, but upregulation of RKIP [70,71,72]. Therefore, a pro- vs. anti-PDT signaling role for endogenous NO was demonstrated based on the level of photodynamic pressure applied. Whether this intriguing discovery also applies to other cancer cell types remains to be seen. In related work, Della Pietra et al. [73] showed that PC3 cells exposed to a modest photodynamic insult utilized iNOS/NO and the NF-κB/Snail/RKIP axis to activate EMT for greater growth and migratory aggressiveness. On the other hand, Huerta et al. [74] reported that DETA/NO at relatively high concentrations can tilt the NF-κB/Snail/RKIP axis of colon cancer cells into the pro-death mode, suggesting that this NO donor might be useful as a PDT adjuvant. However, important issues such as suitable dosage and the possibility of negative side effects have not been addressed.

## 8. Role of iNOS-Derived NO in PDT Bystander Effects

Cancer cells exposed to physical or chemical provocations often send stress signals to unprovoked neighboring cells (bystanders), which may cause the latter to respond in diverse ways, ranging from viability loss to stimulated growth [75]. This phenomenon, referred to as a bystander effect, has been recognized and studied for many years, particularly by ionizing radiation biologists [76,77,78]. However, except for two studies seven years ago [79,80], relatively little bystander work has been done in the context of PDT, which involves non-ionizing radiation. When a PDT photosensitizing agent or metabolic precursor like ALA is administered, not all cells in a given tumor will be equally sensitized. Moreover, during irradiation, the delivered light fluence may vary across the treated tumor area. For these reasons, some groups of tumor cells may be exposed to a significantly greater photodynamic challenge than others, and one can hypothesize that the former can send stress signaling mediators to the latter, acting as bystanders. NO could be one such mediator. Evidence for this was recently reported by Bazak et al. [81], using an innovative approach to distinguish ALA/light-targeted PC3 cells from non-targeted PC3 bystanders. One particularly striking observation in this study was that after irradiation, iNOS and NO levels increased progressively in the bystander as well as targeted cell populations. This suggested that a NO-mediated feed-forward signaling mechanism was in operation [81]. Like surviving targeted cells, bystanders proliferated and migrated at significantly greater rates than ALA-only or light-only controls, iNOS-derived NO being the major driving factor based on strong inhibition by 1400 W and cPTIO. On the other hand, conditioned medium from targeted cell had no effect on bystander growth or migration, discounting any contributions of factors with longer lifetimes than NO, e.g., H_2_O_2_ or cytokines. In initial studies aimed at examining the status of key pro-survival/pro-growth effector proteins in the bystander compartment, it was found that phosphorylation-activation of two kinases, Akt and ERK1/2, was significantly increased relative to controls, while cyclooxygenase-2 (COX-2) was overexpressed [81]. These findings raise important new questions about negative off-target effects in clinical PDT. Because NO from targeted tumor cells could provoke greater aggressiveness in non- or poorly-targeted bystanders, greater tumor expansion over that occurring without PDT might result. This concern could be readily addressed through rational use of iNOS inhibitors, as discussed below.

## 9. iNOS Inhibitors as Potential PDT Adjuvants

Realizing that a number of malignant tumors rely on iNOS/NO signaling for a survival, proliferative, and metastatic advantage, many investigators have advocated pharmacologic use of iNOS inhibitors to curb tumor progression [13,14,15,33]. Such inhibitors might be used alone or in combination with existing chemotherapeutic or radiotherapeutic approaches for possible synergistic effects. Based on the experimental evidence described in preceding sections, the same ideas could easily apply to clinical PDT. Thus, there is good reason to believe that PDT outcomes for a variety of malignancies would be significantly improved through use of select iNOS inhibitors as pharmacologic adjuvants. Reflecting favorably on this is the fact that two such inhibitors, GW274150 and L-NIL, have already been tested in clinical trials unrelated to cancer or PDT (i.e., for controlling asthmatic inflammation) and with no unfavorable side effects [82,83]. Considering that GW274150 has demonstrably improved PDT efficacy in a human tumor xenograft model (see Section 6.1), this inhibitor should be a good candidate for future testing in a clinical trial setting.

## 10. Summary and Conclusions

**Figure 5:**
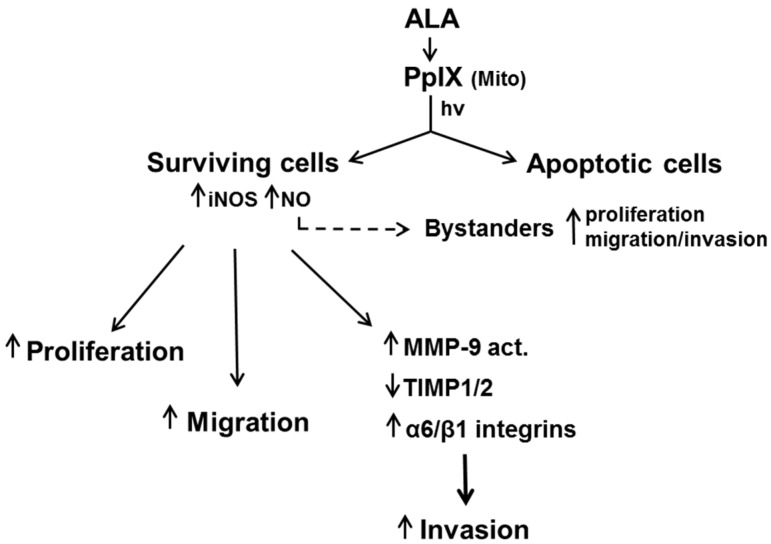
Enhanced NO-mediated growth, migratory, and invasive aggressiveness of cancer cells that survive an ALA-PDT challenge. These responses can apply not only to cells that are direct targets of photodynamic action, but also non-targeted bystander cells which are accessible to NO from targeted cells.

It is now widely recognized that many tumors utilize low flux NO to resist apoptosis, stimulate expansion through accelerated proliferation and migration/invasion, and also to resist eradication by anti-tumor therapies such as PDT. This NO can be generated by tumor cells per se, but surrounding vascular cells such as eNOS-expressing endothelial cells may also contribute. In vitro studies in the author’s laboratory have shown that several cancer cell types, including breast, prostate, and glioma, will rapidly and persistently overexpress iNOS and NO after a PDT-like oxidative challenge. This NO not only signals for greater resistance to photokilling, but also an altered phenotype in surviving cells which is characterized by more aggressive proliferation, migration, and invasion (Figure 5). These changes are underscored by activation/upregulation of effector proteins such as Akt, ERK1/2, COX-2, Survivin, MMP-9, integrins, and S100A4, all of which are associated with tumor promotion/progression [3,4,19,30]. This raises a serious concern because the intensity with which PDT is applied (like that of other anti-tumor treatments) must be carefully modulated in order to minimize damage to normal tissues. As a consequence, tumor elimination is typically not complete and remaining live cells might be more aggressive, as observed with in vitro model systems [56,57,61]. However, the administration of appropriate iNOS inhibitors prior to and after PDT could alleviate such concerns, as discussed in Section 9. Thus, enthusiasm for their eventual entry into the PDT clinic should remain high.
